# Liquid biopsy in triple-negative breast cancer: a promising tool for diagnosis, prognosis, and treatment monitoring

**DOI:** 10.3389/fonc.2025.1642521

**Published:** 2026-01-12

**Authors:** Marianna Rita Brogna, Gerardo Ferrara, Valeria Varone, Angela Montone, Adriana Fava, Maria Rosaria Schiano, Michele Del Sesto, Nubia Pizza, Annalisa Prota, Carmela Barra, Francesca Collina

**Affiliations:** Pathology Unit, Istituto Nazionale Tumori‐Istituto Di Ricerca E Cura A Carattere Scientifico (IRCCS)‐Fondazione G. Pascale, Naples, Italy

**Keywords:** circulating tumor cells (CTCs), circulating tumor DNA (ctDNA), liquid biopsy, precision oncology, treatment response, triple-negative breast cancer (TNBC)

## Abstract

Triple-negative breast cancer (TNBC) is a highly aggressive and heterogeneous subtype of breast cancer, characterized by poor prognosis and limited treatment options. With the advancements in precision oncology, liquid biopsy has emerged as a promising, non-invasive tool for diagnosing TNBC, predicting prognosis, and monitoring treatment response. Circulating tumor DNA (ctDNA) and other biomarkers, such as circulating tumor cells (CTCs), exosomal RNA, and non-coding RNAs, offer valuable insights into tumor heterogeneity, disease progression, and therapeutic efficacy. Recent research has indicated that the presence of ctDNA following neoadjuvant chemotherapy is associated with reduced progression-free survival. Additionally, mutations in key genes such as *TP53* and *PIK3CA/AKT*, along with microRNA alterations (e.g., miR17, miR19a, miR105), have been identified as potential indicators of treatment response and resistance. Furthermore, the study of CTCs enables real-time tumor dissemination profiling, which may enhance prognostic accuracy. Despite these promising developments, significant challenges persist in the standardization and clinical implementation of liquid biopsy techniques. This review provides a comprehensive overview of the current and future applications of liquid biopsy in TNBC management, highlighting its potential to improve early disease detection, optimize therapeutic strategies, and refine patient classification. Integrating liquid biopsy into routine clinical practice could lead to better treatment outcomes and more personalized approaches to TNBC care.

## Highlights

- Each liquid biopsy analyte offers specific insights and is useful for addressing different clinical questions.
- Circulating tumor cells (CTCs) are reliable biomarkers for providing both prognostic and predictive clinical information.
- Advances in CTC analysis have improved our understanding of their role in predicting therapy sensitivity or resistance. Changes in CTC levels during treatment may also serve as a tool for pharmacodynamic monitoring.
- Cell-free DNA (cfDNA) and circulating tumor DNA (ctDNA) are promising biomarkers with strong prognostic and predictive potential in breast cancer.
- Extracellular vesicles (EVs) can be used not only for cancer diagnosis and prognosis but also as innovative vehicles for targeted drug delivery.
- Liquid biopsy can enhance early disease detection, optimize treatment strategies, and refine patient classification in TNBC management.

## Introduction

Breast cancer is the most commonly diagnosed malignancy among women, comprising approximately 25% of all female cancers. Despite substantial progress in diagnostic techniques and therapeutic interventions, it continues to represent the leading cause of cancer-related mortality in the female population (1). In addition to the classical clinical classification based on ER, PR, HER2 status, breast cancer heterogeneity is more accurately captured by the intrinsic molecular subtypes described by Perou et al. This gene-expression–based taxonomy identifies biologically distinct groups with different behaviors and therapeutic vulnerabilities. The luminal A subtype is characterized by strong hormone-receptor signaling and low proliferative activity, and it is generally associated with a more favorable prognosis. Luminal B tumors also express hormone-receptor–related genes but show higher proliferation rates and, in some cases, HER2 activation, resulting in a more aggressive clinical course. The HER2-enriched subtype is defined by pronounced activation of the HER2 pathway and limited hormone-receptor expression, making it particularly responsive to HER2-targeted therapies.

Finally, the basal-like subtype, which largely overlaps with triple-negative breast cancer, displays high proliferation, genomic instability, and poor prognosis. A fifth group, known as normal-like, resembles non-tumoral breast tissue; however, its clinical relevance remains limited and is sometimes considered a technical artifact.

TNBC is more common in younger women, displays a high proliferative index, and is associated with early relapse, poor prognosis, and increased mortality (1, 2). While chemotherapy remains the standard treatment, response rates vary significantly. Approximately one-third of patients achieve a pathological complete response (pCR) with favorable survival outcomes, whereas the majority experience residual disease and an increased risk of recurrence. Metastatic TNBC has particularly poor survival rates, with over 70% of patients succumbing to the disease within five years (2). Chemotherapy remains the primary treatment for TNBC, but many patients do not achieve complete remission, emphasizing the need for targeted therapies. Key biomarkers under investigation include BRCA1/2 mutations, found in 10%-20% of TNBC cases, and PD-L1 expression, detected in 20%-38% of metastatic cases. Elevated tumor-infiltrating lymphocytes (TILs) correlate with better prognosis, improved immunotherapy response, and higher pCR rates. Histopathological examination remains the gold standard for diagnosis, though limitations such as tumor heterogeneity (2, 3). Genomic profiling has uncovered promising therapeutic targets, including DNA damage response pathways, angiogenesis inhibitors, immune checkpoint inhibitors, and anti-androgen therapies, all of which are currently being tested in clinical trials. However, genetic alterations in TNBC can evolve during treatment, potentially rendering driver mutations functionally neutral and contributing to therapeutic resistance. These challenges underscore the urgent need for personalized therapeutic strategies to improve patient outcomes in TNBC. Although combination chemotherapy improves response rates, TNBC remains a major clinical challenge due to the lack of targeted treatments (3). Research continues to explore ways to better characterize and treat this aggressive form of breast cancer. Metastatic TNBC exhibits significant heterogeneity due to various somatic mutations and molecular alterations, necessitating the identification of novel biomarkers to guide treatment and improve clinical management. Tumor evolution and intratumoral heterogeneity contribute to treatment resistance, driven by cellular mutability and selective drug pressures. While serial biopsies and continuous monitoring could provide insights into tumor progression, their invasive nature limits feasibility (3, 4). Advances in liquid biopsy, particularly circulating tumor DNA (ctDNA) analysis, offer a promising alternative for detecting tumor heterogeneity, monitoring disease progression, and assessing treatment response (4) This review examines the clinical relevance of ctDNA and other liquid biopsy components in breast cancer, with a focus on TNBC. It discusses recent advances in noninvasive technologies for prognostic and predictive assessment, highlighting ctDNA’s potential to guide personalized treatment, monitor response, and improve outcomes. The utility of CTCs, exosomes, and RNA-based biomarkers is also addressed, along with future directions for integrating liquid biopsy into routine clinical care.

## Clinical and molecular features of triple-negative breast cancer

TNBC represents around 10-15% of all breast cancer diagnoses. While the overall five-year survival rate is 78.6%, it decreases markedly in advanced stages (III and IV). Treatment is challenging due to the absence of HER2, estrogen, and progesterone receptors, which restricts targeted therapy options. The disease is further complicated by its biological heterogeneity, aggressive nature, and frequent late detection. Moreover, the lack of effective biomarkers hinders early intervention and contributes to unfavorable outcomes (5). In addition to the intrinsic subtypes of breast cancer defined by Perou et al., triple-negative breast cancer (TNBC) itself exhibits substantial molecular heterogeneity. A pivotal study by Jiang et al. analyzed a cohort of 465 primary TNBCs and identified four transcriptome-based subtypes with distinct genomic features and potential therapeutic vulnerabilities.

### Luminal androgen receptor

This subtype is driven by androgen receptor (AR) signaling and shows frequent *ERBB2* somatic mutations and *CDKN2A* loss. Although ER-negative, LAR tumors display high AR expression and may respond to anti-androgen therapies. They are also enriched for *MUC1* and other AR-related genes.

### Immunomodulatory

Defined by strong expression of immune-cell signaling pathways, cytokines, and antigen-presentation machinery, this subtype reflects a highly active immune microenvironment. IM tumors tend to respond more favorably to immune-targeted therapies.

### Basal-like immune-suppressed

Characterized by low immune activation despite a basal-like phenotype, BLIS tumors show suppression of immune-response genes together with upregulation of cell-cycle and DNA-repair pathways. This subtype is associated with a poorer prognosis and is enriched for genes such as *SOX* and *VTCN1*.

### Mesenchymal-like

Enriched for pathways involved in epithelial-to-mesenchymal transition (EMT), cell motility, growth-factor signaling, and stromal interactions. MES tumors display more aggressive biological behavior and exhibit resistance to multiple therapies. This subtype is enriched for genes such as *IGF1* and prostaglandin receptors.

Although not part of the 2019 Cancer Cell four-subtype framework, an additional category—Basal-like Immune Activated (BLIA)—has been described in complementary classifications of TNBC. BLIA tumors exhibit strong immune activation, high expression of immune-related transcription factors (such as *STAT* family genes), and are associated with a more favorable prognosis. Their immunologically active profile suggests potential sensitivity to immune-checkpoint inhibitors and other immunotherapeutic strategies (4–6).

## Genetic and pathological features

Clinical decisions for TNBC still primarily depend on the assessment of three markers: ER, PR, and HER2. Advances in ‘omics’ technologies (such as genomics, epigenomics, transcriptomics, and proteomics) have provided deeper insights into the heterogeneity of TNBC, uncovering potentially actionable molecular features in some subtypes (5, 6). On average, TNBC tumors carry 1.68 somatic mutations per megabase of coding regions, resulting in approximately 60 somatic mutations/tumor. Most of these mutations are present at low (<5%) or very low (<1%) allele frequencies, which makes their detection challenging in routine clinical testing. Among the genes involved, BRCA1 and BRCA2 play a pivotal role in maintaining genomic stability, as they are essential for the homologous recombination repair of DNA double-strand breaks. Loss-of-function mutations in these genes compromise DNA repair, leading to genomic instability and increased susceptibility to tumor development. Detecting such rare mutations accurately is critical for identifying patients who may benefit from targeted therapies. Mutations in these genes are found in about 10% of triple-negative breast cancer (TNBC) cases (6, 7). In cells deficient in BRCA1/2, the inhibition of PARP—an enzyme involved in repairing single-strand DNA damage—leads to the accumulation of DNA breaks, ultimately causing cell death. This mechanism is the basis for the use of PARP inhibitors, such as Olaparib, which significantly improved progression-free survival in BRCA-mutated TNBC patients in the OlympiAD trial, compared to standard chemotherapy (7).

Furthermore, the PrECOG 0105 neoadjuvant trial demonstrated that TNBC patients with BRCA1/2 mutations had the highest response rate (75%) to a regimen including gemcitabine, carboplatin, and the PARP inhibitor iniparib. This highlights the potential benefit of targeting DNA repair deficiencies in this patient population (8).

While over 75% of BRCA1-mutated and about 50% of BRCA2-mutated breast cancers exhibit a TNBC phenotype, only 10–20% of all TNBC cases are due to inherited (germline) BRCA mutations. An additional 30–40% show sporadic BRCA1/2 inactivation, often caused by promoter hypermethylation—a phenomenon referred to as “BRCAness”, which mimics hereditary BRCA-related cancers (9).

TNBC also displays dysregulation in the PI3K/AKT signaling pathway, with activating mutations in PIK3CA and AKT1 found in 25-30% of advanced cases. This suggests that combining AKT inhibitors with chemotherapy could improve treatment outcomes. Additionally, Some TNBCs overexpress PDL1, leading to genomic instability and immune infiltration. PD-L1 inhibitors, such as atezolizumab and pembrolizumab, have shown promising results, with atezolizumab receiving FDA approval for metastatic TNBC in 2019 (10). Basal-like Immune Activated (BLIA) has the best prognosis, while Basal-like Immune Suppressed (BLIS) has the worst. Despite significant progress in the molecular profiling of triple-negative breast cancer (TNBC), a comprehensive classification system that integrates genomic, transcriptomic, and histological data has not yet been established (1, 2, 10). This lack of a unified framework, combined with the biologically aggressive nature of TNBC and the limited availability of effective targeted therapies, complicates its clinical management. As a result, TNBC remains a challenging cancer to treat due to its aggressive behavior, the scarcity of reliable biomarkers, and the absence of personalized treatment strategies. The development of an integrated classification system and the identification of novel actionable mutations are critical for advancing precision medicine and improving patient outcomes (1–4).

Liquid biopsy represents a promising diagnostic tool to improve the management and prognosis of TNBC. However, most current techniques are not specifically optimized for TNBC or basal-like tumors, raising concerns about their detection accuracy in these subtypes. This review explores state-of-the-art mutation- and DNA methylation-based liquid biopsies and evaluates their potential applications in TNBC and basal-like cancers (10).

## Liquid biopsy applications in triple-negative breast cancer

Liquid biopsy has become a promising, non-invasive diagnostic tool for monitoring cancer progression in real-time by analyzing biomarkers like ctDNA and CTCs. ctDNA, in particular, is gaining recognition as a powerful biomarker for improving breast cancer diagnosis, treatment selection, and prognosis. Unlike traditional tissue biopsies, liquid biopsy provides a non-invasive, real-time way to track tumor behavior, detect minimal residual disease (MRD), and identify early signs of recurrence. This is especially valuable for aggressive breast cancer subtypes like TNBC, which have high relapse rates and require precise monitoring (11).

In the context of TNBC, ctDNA analysis holds significant promise for detecting MRD during neoadjuvant chemotherapy, with emerging evidence indicating a correlation between ctDNA levels and patient survival outcomes (12). Particularly in early-stage TNBC, post-neoadjuvant ctDNA assessment has demonstrated potential in predicting recurrence, thereby facilitating earlier and more personalized therapeutic interventions. Compared to traditional biomarkers, ctDNA-based liquid biopsy provides superior sensitivity and clinical utility, especially in recurrent and metastatic settings, where the detection of mutations such as ESR1 and PIK3CA can assist in guiding treatment decisions and evaluating drug response and resistance (13).

Despite promising findings, the clinical use of ctDNA in breast cancer, especially in TNBC, remains limited due to the lack of standardized protocols for detecting recurrence based on genetic alterations (11, 14). Most evidence comes from small-scale retrospective studies. While ctDNA and CTCs show prognostic value, with ctDNA capable of detecting relapse months before clinical signs, routine clinical use is still restricted, particularly for asymptomatic patients (14). Ongoing trials are needed to refine liquid biopsy methods and validate ctDNA’s role in monitoring disease progression and guiding personalized treatments, with a focus on TNBC. Current clinical investigations aim to establish the role of reliable blood-based biomarkers to enhance disease monitoring and improve therapeutic outcomes in breast cancer care (13–15).

### Circulating cell-free DNA

CfDNA is released into the bloodstream either through active secretion by cells or as a result of cellular death processes like apoptosis and necrosis. Its concentration tends to be higher in individuals with advanced cancers compared to early-stage patients or healthy individuals. Within the pool of cfDNA, only a fraction originates from tumor cells — this tumor-specific DNA is known as ctDNA, and its presence can only be confirmed by detecting cancer-related genetic mutations (15). Extensive research has explored both cfDNA and ctDNA as promising biomarkers for prognosis and disease monitoring in various cancers, including breast cancer (BC). The identification of mutations within ctDNA not only helps in distinguishing malignant from benign breast conditions but also holds potential for tracking disease evolution. However, most current studies have concentrated more on refining ctDNA detection techniques rather than establishing their clinical significance for predicting treatment response or survival outcomes (14, 15). A key obstacle in leveraging ctDNA for clinical use is its typically low concentration compared to total cfDNA, even in patients with advanced or metastatic disease, which makes accurate detection challenging. Nonetheless, research has shown that higher ctDNA levels often correlate with greater tumor burden and advanced disease stages. Beyond simple detection, ctDNA mutation profiling has proven helpful for identifying resistance mechanisms to targeted therapies. One study by Ma et al. demonstrated that HER2 gene amplification could be detected via ctDNA in patients who developed resistance to HER2-targeted therapy, while Santonia et al. highlighted cases where HER2 amplification was absent in ctDNA despite therapy resistance, underlining the complexity of interpreting ctDNA data (16, 24).

When compared to other biomarkers like CA 15–3 or CTCs, ctDNA appears to provide a more accurate reflection of a tumor’s genetic landscape and burden. Studies have shown that ctDNA and total cfDNA levels are significantly associated with progression-free and overall survival in breast cancer, particularly in the metastatic setting (17).

Despite these promising findings, inconsistencies in detection techniques, a lack of standardized protocols, and varying definitions between cfDNA and ctDNA continue to limit their full clinical implementation. ctDNA is more specific, as it directly reflects tumor mutations, but is harder and more expensive to detect (18). In contrast, total cfDNA might still offer prognostic value due to its correlation with tumor burden, although it lacks the genetic specificity of ctDNA.

### Circulating tumor DNA in triple-negative breast cancer

In the context of TNBC, multiple studies have demonstrated the potential role of ctDNA as both a predictive and prognostic biomarker. Key measures like the variant allele frequency (VAF) and tumor fraction (TFx) — which represent the proportion of tumor-derived mutations in the blood — have shown strong correlations with disease severity, treatment response, and patient outcomes (17, 18).

Cailleux et al. highlighted the utility of personalized ctDNA monitoring to assess treatment response and disease progression in breast cancer patients, while Park et al. demonstrated that high baseline cfDNA levels could predict relapse in TNBC, even though correlations with imaging responses were limited by sample size (18, 19). Similarly, Radovich et al. reported that the persistence of ctDNA after neoadjuvant chemotherapy (NAC) was linked to a higher risk of relapse and worse two-year survival, reinforcing the value of ctDNA as a post-treatment prognostic tool (19). Wongchenko et al. also confirmed that elevated ctDNA levels during treatment correlated with shorter progression-free survival, particularly in patients with PIK3CA or AKT mutations (20). Further studies, including those by Cavallone et al., showed that ctDNA could detect genomic alterations associated with therapy resistance and tumor progression, including in TNBC (21, 22). ctDNA has also proven useful for assessing changes in copy number alterations, which are common in TNBC and may inform both prognosis and treatment decisions, such as sensitivity to platinum-based chemotherapy and detecting minimal residual disease. However, the implementation of ctDNA-based testing in routine clinical practice still requires standardized methods, large-scale clinical validation, and integration into therapeutic decision-making (19–21).

## The importance of testing for circulating tumor DNA in TNBC

While traditional tissue biopsies remain a cornerstone for cancer diagnosis, they are invasive and provide only a snapshot of the tumor at a single point in time, often failing to capture the full spectrum of genetic heterogeneity. In contrast, cfDNA—particularly the fraction originating from tumor cells, known as ctDNA—offers a non-invasive and dynamic alternative for monitoring disease progression (21). Due to its short half-life, ctDNA reflects the current tumor burden, enabling the early detection of genetic alterations, monitoring of treatment response, assessment of minimal residual disease, and informed therapeutic decision-making in breast cancer. Despite its promise as a biomarker, several technical limitations remain. The reliability of ctDNA analysis is highly dependent on proper sample collection and processing, as mishandling can lead to contamination or reduced sensitivity. For optimal results, plasma is preferred over serum, with rapid processing, appropriate storage conditions, and the use of stabilizing collection tubes being strongly recommended (22) **Table 1**.

**Table 1:** Comparison between tissue biopsy and ctDNA through a series of features.

FEATURES	TISSUE BIOPSY	ctDNA
POSSIBLE ANALYSES	Genomic, Transcriptomic and Proteomic	Genomic
ACCESSIBILITY	Depends on tumor site	Yes
INVASIVENESS	Yes	NO
RISK FOR PATIENT	Depends on tumor site	Low
SAMPLING FAILURE	Depends on tumor site	Low
IMPORTANCE OF SAMPLING TIME	No	Low ctDNA yield while receiving efficient therapy
COST	high	Low
PROCEDURES	standardized	not standardized
USE OF HIGH SENSITIVE DETECTION METHODS	not necessary	depends of tumor burden(which influences ct DNA yield)
IMPORTANCE OF TUMOR CELLULARITY	intermediate	high
NEED OF IMMEDIATE PROCESSING	Depends on the analysis	Requires plasma isolation or the use of streak tube collection
STABILITY AFTER PROCESSING	Depends on the analysis	yes
CAPTUR OF INTRA AND INTER TUMOR HETEROGENEITY	no	yes
LONGITUDINAL MONITORING OF THE DISEASE	hardly acceptable by patients	multiple assessment possible

## ctDNA detection in TNBC: PCR and NGS-based strategies

Detection methods are broadly categorized into PCR-based and next-generation sequencing (NGS)-based techniques, each with specific advantages and limitations. Emerging ctDNA assays are classified into two categories: tumor-informed and tumor-agnostic assays (23).

- Tumor-informed assays leverage specific genetic alterations identified through tumor tissue sequencing to customize the ctDNA assay for the individual patient. This approach ensures that the ctDNA analysis is directly tailored to the unique mutations of the patient’s tumor, offering greater precision in detecting tumor-specific genetic alterations in the bloodstream.
- Tumor-agnostic assays, in contrast, focus on broader genomic, epigenomic, or fragmentomic patterns to detect ctDNA without prior knowledge of specific mutations. This methodology is beneficial in cases where mutations may be unknown, heterogeneous, or not yet well-characterized, and it facilitates detection across a range of tumor types (24). A tumor-agnostic assay employs a standardized panel that can be applied to any patient without requiring prior knowledge of the tumor’s genetic profile.

PCR-based techniques—such as ARMS-PCR, ddPCR, and BEAMing—offer extremely high sensitivity, capable of detecting mutant alleles at frequencies as low as 0.001% (1 in 100,000 wild-type molecules). However, their major limitation lies in requiring prior knowledge of the target mutation, which restricts their utility in tumors with poorly characterized genetic profiles. In breast cancer studies, particularly in metastatic settings, PCR-based detection of mutations in *PIK3CA* and *TP53* has shown good concordance with tissue-based findings and has been correlated with treatment response and disease progression. Notably, ddPCR has also enabled the detection of tumor-specific chromosomal rearrangements, with detection limits around 0.01% (25).

In contrast, NGS-based approaches allow for broader mutational screening, particularly useful in identifying non-recurrent or novel mutations such as those in *TP53* or *PTEN (*25, 26). Techniques like *Techniques like tagged-amplicon deep sequencing* (TAm-Seq) and Safe-Sequencing System (Safe-SeqS) improve the analytical sensitivity of NGS by minimizing sequencing errors and enabling reliable detection of ctDNA even at low allelic fractions (<1%). Recent studies in TNBC patients have demonstrated a high concordance between tumor and plasma DNA for TP53 mutations, with mutant allele fractions ranging from 2% to 70% (median 5%). However, NGS sensitivity remains insufficient for clinical application in early-stage or minimal residual disease, where ctDNA concentration is typically low (26).

**Figure 1:**
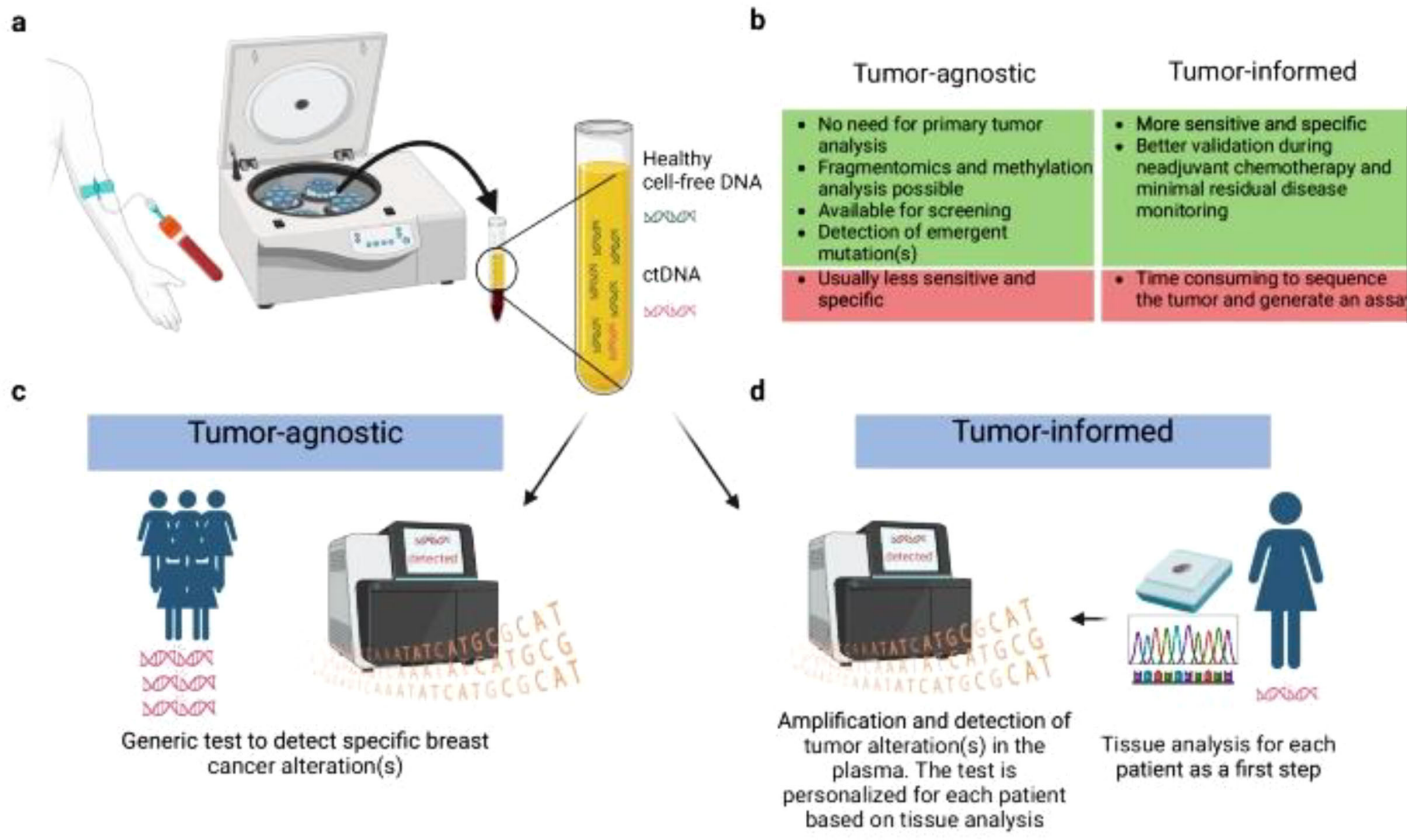
Schematic representation of tumor-agnostic and tumor informed circulating tumor DNA (ct DNA) analysis: **(A)** Ct DNA analysis begin with centrifugation of the blood sample to isolate plasma, followed by DNA extraction **(B)** Advantages and disadvantages of tumor agnostic and tumor informed assays **(C)** A tumor-agnostic assay employs a standardized panel that can be applied to any patient without requiring prior knowledge of the tumor’s genetic profile. While different platforms exist, they generally perform the same type of analysis, though with varying performance characteristic **(D)** In contrast, a tumor-informed ctDNA **assay** involves a two-step process. First, the primary tumor is analyzed—typically through sequencing—to identify specific somatic alterations. Then, customized primers or probes are designed targeting those mutations. In the second step, the plasma-derived DNA is tested to detect the presence of those tumor-specific mutations.

Importantly, in TNBC patients with residual disease after neoadjuvant chemotherapy (NAC), ctDNA detection via NGS has been associated with increased relapse risk. In a cohort of early-stage TNBC patients, post-treatment ctDNA positivity was predictive of disease recurrence, highlighting the potential of ctDNA as a biomarker for risk stratification and treatment tailoring in the post-NAC setting (25, 26) (**Figure 1**).

## Tumor-derived components in liquid biopsy: CTCs, TEPs, exosomes, and ctNAs

Molecular studies are opening new avenues for the clinical management of TNBC, with circulating cell-free tumor nucleic acids (ctNAs) representing one of the most promising research areas.

A major advancement in the clinical application of ctNAs is the possibility of isolating them through liquid biopsy, a non-invasive procedure that analyzes various biological fluids — including blood, urine, cerebrospinal fluid, and pleural effusion — for tumor-derived components (27).

Liquid biopsy allows the detection and monitoring of cancer in real time by isolating CTCs, circulating cell-free nucleic acids (ccfNAs), exosomes, and tumor-educated platelets (TEPs). These components reflect the genetic and phenotypic heterogeneity of both primary tumors and metastatic lesions, making them valuable as predictive and prognostic biomarkers (28).

- CTCs are tumor cells that detach from the primary tumor and enter the bloodstream. Despite their extremely low abundance (fewer than 10 cells per mL of blood), they offer important histological, molecular, and functional insights.
- TEPs are platelets that internalize tumor-derived mRNA and can undergo tumor-specific splicing events. They can also sequester soluble tumor-associated proteins, making them potential markers of tumor progression (27, 28).
- EXOSOMES are extracellular vesicles containing nucleic acids and proteins reflective of their originating cells. Their molecular cargo provides important clues about tumor status and behavior.
- ctNAs — the fraction of ccfNAs derived specifically from tumor cells — include circulating tumor DNA (ctDNA), mRNA (ctmRNA), microRNA (ctmiRNA), and non-coding RNAs (ctncRNA). Changes in ctDNA levels and mutation profiles correlate with pathological features such as mutational burden, metastatic spread, and therapeutic response. In addition, the integrity of circulating DNA has been suggested as a diagnostic and prognostic biomarker. Similarly, ctmiRNAs are involved in tumor growth, chemoresistance, and subtype specificity, making them attractive candidates for predictive and prognostic applications (28).

**Figure 2:**
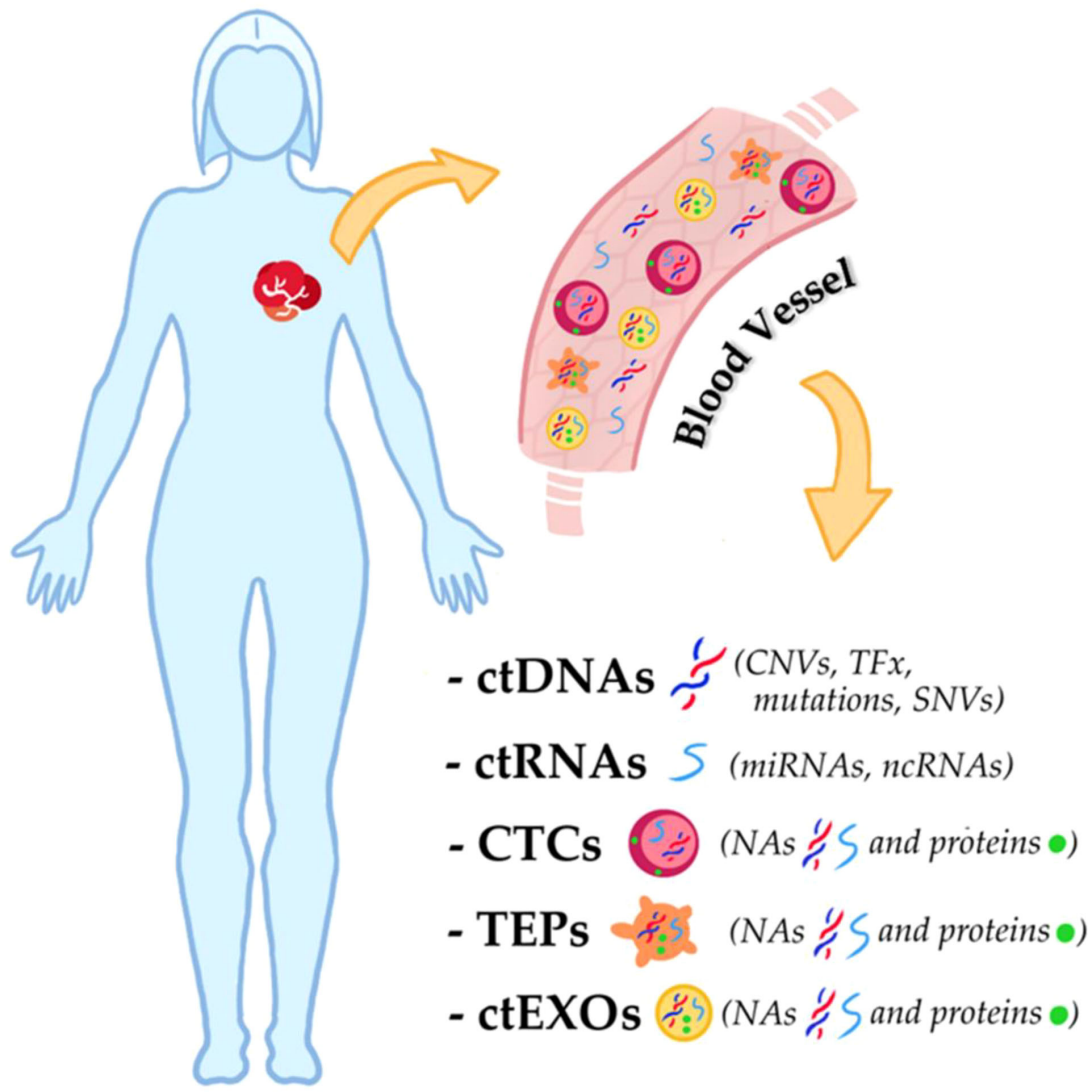
Liquid biopsy biomarkers. As shown in the figure, the biomolecular analysis from body fluids, such as blood, is not limited to circulating cell-free nucleic acids but also considers the tumor biomolecules carried by CTCs, TEPs, and ctEXOs A variety of circulating biomarkers—including circulating tumor cells (CTCs), circulating tumor DNA (ctDNA), cell-free RNA (cfRNA), and extracellular vesicles (EVs)—can enter the bloodstream from both primary tumors and metastatic sites. The schematic illustrates the principle of liquid biopsy: blood samples collected from cancer patients contain these tumor-derived components, which offer real-time insights into tumor dynamics, prognosis, and response to therapy. **CNVs***, copy number variations;***CTCs,***circulating tumor cells; ctDNAs, circulating cell-free tumor DNAs;***ctEXOs,***circulating tumor exosomes; ctncRNAs, circulating cell-free tumor non-coding RNAs;***NAs,***nucleic acids;***ncRNAs,***non-coding RNAs; SNVs, single nucleotide variations;***TEPs,***tumor-educated platelets;***TFx,***tumor fraction of circulating cell-free DNA.*.

Despite their potential, ctNAs are typically present in low concentrations and are often highly fragmented, which poses technical challenges for clinical use. Therefore, the development of sensitive and specific analytical platforms, such as digital droplet PCR and NGS, as well as optimized workflows for ctNA isolation and preservation, are essential for their reliable use in routine clinical practice (**Figure 2**).

## RNA liquid biopsy targets: circulating cell-free tumor non-coding RNA in TNBC

Non-coding RNAs (ncRNAs) are increasingly recognized as key regulators of gene expression and cancer progression (28, 29). Despite lacking protein-coding capacity, ncRNAs — including microRNAs (miRNAs), long non-coding RNAs (lncRNAs), circular RNAs (circRNAs), and PIWI-interacting RNAs (piRNAs) — modulate transcriptional, post-transcriptional, and epigenetic processes. These molecules influence fundamental cellular behaviors such as proliferation, differentiation, apoptosis, and immune escape. Aberrant ncRNA expression has been implicated in tumorigenesis across various cancer types, including breast cancer (BC), where they are now under active investigation as potential diagnostic and prognostic biomarkers (29).

Among ncRNAs, microRNAs (miRNAs) have drawn particular attention for their potential role in liquid biopsy applications. Tumor-derived miRNAs can be released into the bloodstream, reflecting molecular alterations in cancer cells and offering a non-invasive window into tumor dynamics (29, 30). Their remarkable stability in circulation, coupled with the ability to capture information from various body fluids, positions miRNAs as promising biomarkers for cancer detection and monitoring.

### Circulating tumor-derived microRNAs in TNBC

Preclinical studies have shown that specific microRNA (miRNA) signatures are dysregulated in TNBC, both in patient serum and cell models. These patterns differ from healthy individuals and vary by breast cancer subtype. Unique miRNA profiles were also found in PBMCs, likely influenced by the tumor microenvironment (29).

Circulating microRNAs (ctmiRNAs) are promising prognostic markers in TNBC, with serum miRNA patterns linked to metastasis and survival. Tumor-derived miRNAs (ctmiRNAs) may aid in early diagnosis, subtype classification, and outcome prediction. However, clinical application is limited by study variability and a lack of standardized detection methods (30). For example, Niedzwiechi et al. reported reduced levels of ctmiR-200c in the plasma of TNBC patients compared to hormone receptor–positive breast cancers, consistent with the observed downregulation of miR-200c in TNBC tumor tissues. This miRNA has been linked to the regulation of apoptosis and metastatic potential through modulation of PTEN and TP53 pathways (30).

Expanding on these findings, Qattan et al. identified a TNBC-specific ctmiRNA signature characterized by the upregulation of miR-19a, miR-19b, miR-25, miR-22, miR-93, and miR-210, alongside the downregulation of miR- -93, and miR-210 were associated with worse overall survival, highlighting their potential as 199a. These miRNAs are involved in critical signaling networks that govern proliferation, chemoresistance, immune evasion, and survival. Notably, elevated circulating levels of miR-19a, miR-19b, miR prognostic biomarkers (29–32).

Similarly, Li et al. demonstrated significant overexpression of miR-93 and miR-105 in TNBC patients, both of which are known activators of the Wnt/β-catenin pathway. Activation of this pathway is associated with enhanced cancer stemness, metastatic dissemination, and therapy resistance—features that underpin the aggressive clinical behavior of TNBC (30). High circulating levels of these miRNAs were predictive of poor clinical outcomes. These findings were further corroborated by a machine learning–based analysis aimed at identifying a TNBC-specific ctmiRNA signature. The analysis highlighted upregulation of miR-17, miR-133b, miR-210, miR-146b, and miR-7, along with downregulation of miR-150 and miR-372. Notably, miR-17, miR-150, and miR-210 exhibited strong discriminatory capacity for both TNBC subtype stratification and prognostic prediction. Additionally, Ritter et al. developed a serum- and urine-based ctmiRNA panel for TNBC detection, highlighting differential expression patterns across both biofluids (33). Increased serum levels of let-7a, let-7e, and miR-21, alongside reduced levels of miR-15a, miR-17, miR-18a, miR-19b, and miR-30b, were observed in TNBC patients. Urine analysis further confirmed the downregulation of miR-18b, miR-19b, miR-30b, miR-222b, and miR-320c, underscoring the potential of ctmiRNAs as versatile, non-invasive biomarkers (31, 33).

### Long non-coding RNAs and other ncRNAs in TNBC

Beyond miRNAs, long non-coding RNAs (lncRNAs) have also demonstrated significant diagnostic and prognostic relevance in TNBC (33). Wang et al. reported that plasma levels of the lncRNA TINCR were markedly elevated in TNBC patients, with high TINCR expression correlating with shorter overall and relapse-free survival. In parallel, Ruiz et al. identified hypermethylation of the lncRNA LINC00299 as a candidate early detection marker, particularly in younger patients with TNBC (33, 34). Notably, the hypermethylated region within LINC00299 contains transcription factor binding sites implicated in immune regulation and tissue homeostasis, suggesting a mechanistic link between its dysregulation and tumor biology. Taken together, these findings emphasize the growing relevance of circulating ncRNAs, particularly ctmiRNAs and lncRNAs, as promising biomarkers for TNBC diagnosis, prognostication, and therapeutic stratification. Despite encouraging results, further validation in large-scale, prospective clinical trials, as well as assay standardization, will be essential before their integration into routine oncologic care (34).

## Extracellular vesicles as potential biomarkers in triple-negative breast cancer

Extracellular vesicles (EVs), including *exosomes and microvesicles*, are membrane-bound structures secreted by cells that encapsulate a broad spectrum of bioactive molecules such as DNA, miRNAs, mRNAs, proteins, and lipids. These vesicles are pivotal mediators of intercellular communication and contribute to key oncogenic processes including metastasis, angiogenesis, immune evasion, and therapy resistance. In TNBC, tumor-derived exosomal miRNAs—particularly miR-21, miR-1246, and miR-939—have been implicated in tumor aggressiveness, lymph node involvement, and disease-free survival, highlighting their potential as prognostic biomarkers (35). Recent studies and meta-analyses underscore the diagnostic and predictive utility of exosome-associated molecules in breast cancer. Circulating exosomes, owing to their remarkable stability in bodily fluids, represent a valuable substrate for liquid biopsy approaches. Exosomal cargo, including tumor-associated proteins (e.g., CEA, CA15-3) and distinct miRNA signatures, mirrors the molecular landscape of the primary tumor and has shown correlations with tumor subtype, response to neoadjuvant chemotherapy, and risk of recurrence in TNBC. While ctDNA remains the gold standard for genomic mutation profiling due to its higher sensitivity and specificity, exosomal biomarkers provide complementary insights and are actively investigated for their clinical applicability in TNBC diagnostics, prognostication, and therapeutic monitoring (36).

## ctDNA in early stage TNBC

In the early stages of breast cancer (BC), when the malignancy remains confined to the breast and regional lymph nodes, ctDNA levels in the bloodstream are generally low, reflecting a limited tumor burden. This poses a challenge for ctDNA detection, particularly in TNBC, where the genomic landscape is frequently undefined. For this, broad genomic strategies — including targeted sequencing panels and whole-exome sequencing (WES) — are recommended in these cases, provided that the assay sensitivity is adequate to detect low-abundance ctDNA (37).

Despite the anatomical accessibility of breast tumors for percutaneous biopsy, recent evidence has highlighted the clinical relevance of ctDNA analysis in early-stage disease. For example, PIK3CA mutations, commonly observed in breast cancer, were successfully detected in plasma samples with high specificity and sensitivity. A study involving 29 patients reported a 93% concordance between PIK3CA mutations detected in tumor tissues and matched pre-surgical plasma samples, underscoring the technical feasibility of ctDNA detection using digital droplet PCR (ddPCR) in early disease (38).

In addition, TP53 mutations are frequently associated with BRCA1-mutated breast cancers. The ongoing prospective CirCA01 trial aims to evaluate the potential of ctDNA as an early diagnostic and surveillance biomarker in asymptomatic BRCA1 mutation carriers. Specifically, the study investigates whether serial monitoring of TP53 mutations in plasma can enable early detection of malignancies, predict relapse, and distinguish new primary tumors from recurrent disease.

In early TNBC, both ctDNA and circulating tumor cells (CTCs) have demonstrated prognostic and predictive value, particularly for early identification of minimal residual disease (MRD) and relapse risk following neoadjuvant chemotherapy (NAC). The Q-CROC-03 trial showed that the persistence of ctDNA after NAC was independently associated with an increased likelihood of relapse, while ctDNA clearance was correlated with favorable long-term outcomes comparable to patients who achieved pCR (39).

Similarly, the I-SPY 2 trial demonstrated that ctDNA persistence following neoadjuvant chemotherapy is significantly correlated with disease recurrence, while ctDNA clearance predicts favorable prognosis (40).

These findings support the potential role of ctDNA as a non-invasive tool for early risk stratification and for tailoring post-treatment management strategies. These observations highlight the potential of ctDNA-based assays to refine therapeutic decision-making and personalize patient monitoring. However, technical limitations, particularly in early-stage disease, and the lack of standardized clinical implementation still pose significant challenges for routine use.

### Detection of MRD

Multiple studies have shown that the presence of ctDNA after NAC is strongly associated with a higher risk of disease relapse, often months before clinical detection (39–41).

Early dissemination of BC cells to secondary sites, including bone marrow and distant organs, may underlie the persistence of minimal residual disease (MRD) in patients without radiological or clinical evidence of metastasis. The prognostic relevance of MRD has been extensively investigated through the detection of disseminated tumor cells (DTCs) in bone marrow and CTCs in peripheral blood, both of which have demonstrated independent associations with metastatic relapse and reduced survival (40).Several studies have explored the clinical significance of ctDNA in this setting. Garcia-Murillas et al. demonstrated the utility of tumor-informed ctDNA assays for MRD detection in early BC, reporting a high concordance between mutations identified in the primary tumor and those detected in plasma samples (41). Longitudinal monitoring revealed ctDNA positivity preceding clinical relapse by a median of 13.6 months. Similarly, Beaver et al. validated the detection of PIK3CA-mutated ctDNA in early-stage BC patients following surgery. Persistent ctDNA was identified in 50% of cases, and its presence correlated with disease recurrence in a subset of patients, including a TNBC case with ctDNA positivity post-chemotherapy who developed distant metastases (41). In the *cTRAK TN trial*, early ctDNA detection was linked to a high rate of metastatic recurrence. However, due to limitations in the timing of blood sample collection and assay sensitivity, ctDNA monitoring did not yet allow for early enough detection to influence treatment decisions. This highlights the urgent need to optimize both sampling schedules and detection technologies, as well as the development of more effective treatments for micrometastatic disease. These observations suggest that ctDNA analysis offers a sensitive and non-invasive approach for early detection of molecular relapse and may enable risk stratification and real-time therapeutic intervention. Nonetheless, prospective clinical trials are warranted to establish its predictive value and clinical applicability in the MRD setting (41, 42).

### ctDNA and response to neoadjuvant chemotherapy in early-stage triple-negative breast cancer

The integration of ctDNA analysis into clinical practice holds significant promise for improving the management of TNBC, particularly in the neoadjuvant setting. Unlike conventional imaging or tissue biopsy, ctDNA offers a minimally invasive, real-time assessment of tumor dynamics, enabling early detection of therapeutic resistance and potential relapse. Its short half-life allows for timely monitoring of molecular changes, which is critical given the typically suboptimal response of TNBC to neoadjuvant chemotherapy. Evidence suggests that ctDNA can precede radiological detection of metastasis, offering a window for early intervention. Although studies such as that of Turner et al. have highlighted the predictive value of ctDNA—particularly TP53 mutation tracking—conflicting findings from other cohorts underscore the need for broader validation and standardization (43). Overall, while ctDNA represents a promising tool for personalized therapy adaptation and disease monitoring, its routine clinical implementation in TNBC requires further refinement and prospective validation in larger, multicenter studies.

### CTCs and ctDNA in metastatic TNBC

In patients with metastatic breast cancer, especially TNBC, the levels of ctDNA in the bloodstream are generally higher compared to early-stage disease (43, 44). This makes ctDNA easier to detect and reduces the need for extremely sensitive testing techniques. As a result, ctDNA has become a valuable clinical tool for early detection of metastatic progression, for providing prognostic insights before starting therapy, and for guiding personalized treatment strategies in metastatic cases (44).

CTCs also play an important role in TNBC prognosis. The presence of CTCs — especially when they form multicellular clusters — is associated with a higher risk of metastasis and poorer clinical outcomes. Research has shown that patients with more than 5 CTCs per 7.5 ml of blood before treatment, or patients whose CTC levels remain high during therapy, tend to have shorter progression-free survival and overall survival. Furthermore, an increase in CTC levels shortly after treatment begins is linked to an unfavorable prognosis.

Despite this, clinical trials like SWOG S0500 have shown that changing treatment early based solely on CTC count does not necessarily improve outcomes (45). Still, many retrospective studies confirm that high baseline CTC levels are a strong indicator of poor prognosis.

Beyond their prognostic value, CTCs can also reveal molecular characteristics of tumors, including genetic mutations such as those in the PIK3CA gene. These mutations sometimes differ from the primary tumor due to tumor evolution and heterogeneity. In fact, the genetic profiling of CTCs helps uncover hidden tumor diversity, which is especially relevant in TNBC, where features like epithelial-to-mesenchymal transition (EMT) and stemness markers are connected to chemotherapy resistance and metastasis (46).

Similarly, ctDNA has become an essential tool for real-time monitoring of disease evolution and therapeutic response in metastatic TNBC. For example, a ctDNA fraction above 10% has been linked to worse clinical outcomes, independently of other clinical factors. Genetic analysis of ctDNA has also identified mutations in key genes, such as TP53 and BRCA1, both of which are closely associated with increased risk of recurrence and poor survival (47).

In addition to detecting known cancer drivers, ctDNA analysis helps identify actionable mutations and informs treatment choices, especially when using NGS. Mutations in genes like BRCA1/2, TP53, PIK3CA, FGFR1/2, ERBB2 (HER2), ERBB3, and the PI3K/AKT/mTOR pathway have all been identified in ctDNA from TNBC patients, offering opportunities for personalized therapy, including the use of PARP inhibitors and targeted drugs (46, 47).

## Potential of ctDNA for early detection and confirmation of metastatic relapse in TNBC

**Figure 3:**
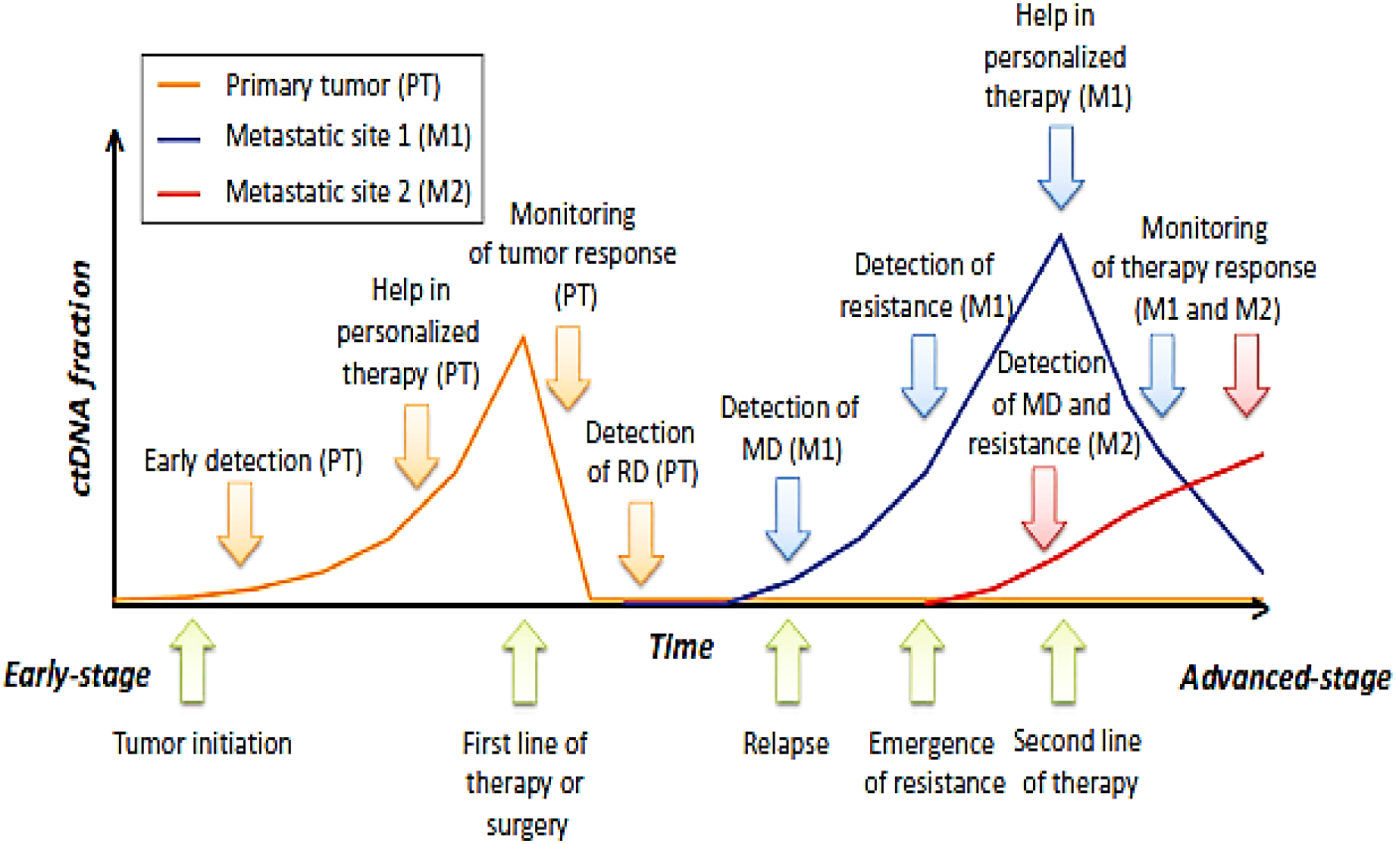
Potential application of circulating tumor DNA (ct DNA) in the management of triple negative breast cancer From early to advanced stages of disease progression (with green arrows indicating the key clinical milestones for a given patient), the orange, blue, and red curves represent the dynamics of ctDNA carrying genomic alterations originating from the primary tumor (PT), metastatic site 1 (M1), and metastatic site 2 (M2), respectively. The detection and quantification of ctDNA have a wide range of clinical applications at each disease site—primary tumor (orange arrow), first metastatic site (blue arrow), and second metastatic site (red arrow)—including cancer diagnosis, guiding therapeutic decisions, monitoring treatment response, identifying residual disease (RD), detecting metastatic progression (MD), and assessing the emergence of therapy resistance.

Metastatic TNBC is typically diagnosed after the emergence of new clinical symptoms or through imaging when metastases have become sufficiently advanced. Detecting metastasis at an earlier stage could significantly enhance the prognosis for patients with stage IV TNBC(). Olsson et al. highlighted the potential of ctDNA analysis for the earlier detection of metastatic disease in patients who had previously undergone treatment for early-stage BC (48). As previously mentioned, chromosomal rearrangements were successfully identified by ddPCR in plasma samples, on average, 11 months before the first clinical signs of metastasis appeared. Additionally, ctDNA could serve as a reliable tool to confirm metastatic relapse when imaging fails to definitively detect metastases. This approach would negate the need for a solid biopsy, as the mutation responsible for metastasis development would have been identified in prior tumor analysis. However, further studies are required to validate the full potential of ctDNA in such diagnostic application (48, 49) (**Figure 3**).

## DNA methylation as a non-invasive biomarker for early detection of triple-negative breast cancer

Identifying abnormal DNA methylation signatures through non-invasive methods offers a promising avenue for the early detection of TNBC, paving the way for earlier clinical intervention and more tailored therapeutic approaches (50).

In several studies, genome-wide DNA methylation data were analyzed from TNBC tumor tissues, normal breast tissues, peripheral blood samples of TNBC patients and healthy controls, as well as reference samples of sorted blood and mammary cells.

Through stringent selection, verification, and external validation, the researchers identified 23 differentially methylated regions (DMRs) associated with TNBC. A machine learning algorithm was then used to prioritize the most promising DMRs, and six were selected for further evaluation in plasma-derived cell-free DNA (cfDNA) samples (50, 51).

Using droplet digital PCR, the presence of methylation at these regions was confirmed in cfDNA samples from TNBC patients compared to healthy controls. A combined methylation score based on three specific DMRs (associated with the SPAG6, LINC10606, and TBCD/ZNF750 genes) showed the strongest potential for discriminating TNBC patients from controls, achieving an AUC of 0.78 in the test set and 0.74 in the independent validation set (52).

These findings suggest that cfDNA methylation signatures may serve as sensitive and specific non-invasive biomarkers for the early detection of TNBC, highlighting the clinical potential of liquid biopsy for cancer screening, diagnosis, prognosis, and therapeutic monitoring.

However, the authors emphasize that further large-scale and independent studies are warranted to validate these biomarkers before clinical implementation.

### The role of *DNMT1* in triple-negative breast cancer

Epigenetic alterations, especially DNA methylation, play a key role in TNBC development and progression. DNA methylation, primarily mediated by DNA methyltransferases (DNMTs), silences tumor suppressor genes by hypermethylating CpG islands in their promoter regions. Among DNMTs, *DNMT1* is particularly important for maintaining these methylation patterns during cell division and has been directly linked to tumorigenesis (53).

DNMT1 is overexpressed in TNBC and its high expression is associated with poor patient survival. In TNBC, DNMT1 promotes oncogenesis by:

1. Repressing ER expression via promoter hypermethylation.
2. Facilitating epithelial-mesenchymal transition (EMT), a key process in metastasis.
3. Inducing autophagy, which supports cancer cell survival under stress.
4. Promoting cancer stem cell properties, enhancing tumor aggressiveness.

In addition, *DNMT1* is involved in silencing multiple tumor suppressor genes, microRNAs, and epithelial markers that normally inhibit EMT (53, 54). Given its central role in TNBC progression, *DNMT1* represents a promising therapeutic target. Hypomethylating agents (HMAs) such as azacitidine, decitabine, and guadecitabine have shown potential anti-tumor effects and may even improve responses to immune checkpoint inhibitors in TNBC.

Overall, DNMT1-targeted therapies could suppress tumor growth, prevent metastasis, and help overcome the aggressive nature of TNBC. Further research into DNMT1 inhibition could contribute to the development of novel therapeutic strategies for this difficult-to-treat breast cancer subtype (54).

### *DNMT1* polymorphisms and expression in triple-negative breast cancer

Single nucleotide polymorphisms (SNPs) in the DNMT1 gene have been linked to an increased risk of developing TNBC. Case-control studies have shown that specific DNMT1 variants — particularly the T allele of rs2288349 and the C allele of rs16999593 — are significantly associated with higher susceptibility to TNBC, increasing risk by more than four- to five-fold (55, 56). While these SNPs do not appear to influence DNMT1 protein expression directly, they may affect the protein’s function or structure, contributing to cancer development.

Beyond genetic variation, DNMT1 is also overexpressed in TNBC and correlates with poor clinical outcomes. DNMT1, along with DNMT3A and DNMT3B, is frequently upregulated in breast cancer tissues compared to normal breast tissue. High DNMT1 expression is especially prevalent in high-grade tumors, tamoxifen-resistant cancers, and TNBC subtypes, and it is linked to shorter overall survival (OS) and disease-free survival (DFS).

At the molecular level, DNMT1 overexpression promotes hypermethylation of tumor suppressor gene promoters (including ERα and BRCA1), which leads to their silencing and enhances breast cancer aggressiveness, particularly in ER-negative and HER2-positive tumors. Despite frequent co-overexpression of DNMT family members in breast cancer, the regulation of each DNMT appears to be independent and subtype-specific (56).

Therefore, both genetic variations and overexpression of DNMT1 are associated with increased TNBC risk and poor prognosis, underscoring DNMT1’s potential as both a prognostic biomarker and therapeutic target in TNBC.

## Immunological targets and liquid biopsy in triple-negative breast cancer

Identifying reliable tumor-derived predictive biomarkers for triple-negative breast cancer (TNBC) patients receiving immunotherapy remains a significant challenge. Emerging approaches—such as monitoring T-cell clonal expansion and analyzing ctDNA—are showing potential. Meanwhile, the prognostic relevance of CTC quantification, especially in relation to PD-L1 expression on CTCs, is currently under active investigation (57).

Tumor Mutational Burden (TMB), which reflects the number of somatic mutations, could serve as a marker for neoantigen production and T-cell infiltration. In breast cancer (BC), TMB’s role in tumor immunogenicity is uncertain, as it is typically low to intermediate, with TNBC showing the highest median TMB (57, 58). While preliminary data suggests that hypermutated BC may benefit from PD-1 inhibitors, TMB-H is not consistently predictive for immune checkpoint inhibitors (ICIs) across cancer types. Only a small percentage of TNBC cases (8-10%) exhibit high TMB. Researchers propose refining TMB’s predictive value in TNBC by considering specific mutational signatures and other genomic alterations.

Liquid biopsy-based TMB is under investigation, and ctDNA analysis could serve as a clinically relevant biomarker. In various trials, ctDNA molecular response has been linked to survival, with ctDNA dynamics showing stronger correlations. The NIMBUS trial showed that HER2-negative mBC patients with TMB-H benefited from nivolumab plus ipilimumab, with certain patients showing higher response rates, emphasizing the need for optimized TMB cut-off values (58). The SAFIR02 BREAST IMMUNO study identified CD274 (PD-L1 gene) as a new biomarker linked to better responses from ICIs in metastatic TNBC, suggesting new opportunities and challenges for liquid biopsy applications (59). The tumor microenvironment in breast cancer (BC) is characterized by a complex interplay of immunoregulatory molecules and infiltrating immune cells, offering insight into the host’s antitumor immune activity. In triple-negative breast cancer (TNBC), liquid biopsy technologies are proving instrumental in uncovering spatial heterogeneity of PD-L1 expression between primary and metastatic lesions.

Elevated PD-L1 levels on CTCs have been correlated with shorter overall survival in patients with metastatic breast cancer (mBC). Additionally, the presence of immunosuppressive factors such as early myeloid-derived suppressor cells (eMDSCs) and their associated proteins has been implicated in suboptimal responses to neoadjuvant chemotherapy in TNBC.

Additionally, prior studies across all BC subtypes reported elevated levels of CD117+ and CD11b+ granulocytes at diagnosis, which decreased after tumor removal by surgery and radiotherapy — supporting the link between tumor burden and measurable immune response (58–60).

A reduction in serum concentrations of MDSCs, PD-L1-expressing T lymphocytes, and regulatory T cells (Tregs) has been correlated with clinical benefit in BC.

Moreover, tumor-associated macrophages (TAMs), particularly CD163-positive subsets, play a crucial role in tumor progression and immune evasion. Monitoring circulating TAM levels could represent a future serum-based immune biomarker to assess treatment response and potentially guide macrophage-targeted therapies in TNBC patients (60).

## Discussion

TNBC is an aggressive subtype with poor prognosis and limited treatment options. Often diagnosed at an advanced stage, it highlights the urgent need for reliable, non-invasive biomarkers to enable early detection, guide therapy, and improve patient outcomes (1, 61).

This narrative review confirms the prognostic value of ctDNA in early TNBC, particularly around neoadjuvant therapy (NAT). ctDNA detection—especially after NAT or surgery—is consistently associated with worse disease-free and overall survival, identifying patients at high risk of relapse (40, 62). Studies by Garcia-Murillas, and Nader-Marta emphasize the value of ctDNA for early risk stratification and personalized treatment decisions. While pCR remains important, early ctDNA clearance during NAT—regardless of pCR—is linked to better outcomes (62).

Compared to CTCs, ctDNA is more sensitive and easier to detect, making it a more reliable marker of minimal residual disease (MRD). Nonetheless, limitations remain. Some studies, such as those by Chen et al. (2024), Cavallone et al. (2020), and the TBCRC 030 trial, report inconsistent predictive accuracy, including false positives and undetectable ctDNA in relapsing patients. Technical variability, low ctDNA shedding in early disease, and lack of assay standardization also hinder its reliability (21, 63).

These conflicting findings are largely due to technical limitations and differences in patient populations. In early TNBC, ctDNA levels are often very low, making detection difficult. For example, Parsons et al. reported a median ctDNA allele fraction of just ~3.7×10^–4 post-NACT, with 58% of patients having levels below assay sensitivity. Thus, “undetectable” often reflects test limitations rather than true absence of disease. Conversely, detectable ctDNA may originate from small residual clones that can still be eliminated by adjuvant therapies. Despite these challenges, ctDNA provides additional prognostic information beyond traditional markers like pCR. In particular, ctDNA status before and after surgery correlates with pCR and residual cancer burden (RCB), refining risk assessment. Ongoing trials (e.g., CUPCAKE and Apollo) are evaluating ctDNA-guided strategies, though their effectiveness will depend on available therapeutic options (64, 65).

Treatment context also matters. Many early TNBC patients now receive post-NACT therapies like capecitabine or immunotherapy, which improve outcomes even in ctDNA-positive cases. In fact, pooled data show that non-pCR patients now reach 62–70% five-year disease-free survival with modern adjuvant treatment—suggesting that ctDNA positivity no longer reliably predicts relapse on its own. The I-SPY2 trial and Cailleux et al. further support early ctDNA clearance (within three weeks of NAT) as a predictor of pCR and reduced RCB (66). This suggests a role for dynamic ctDNA monitoring in real-time treatment adaptation. Future improvements—such as using multiple gene targets in ddPCR—could enhance detection sensitivity. Specific ctDNA alterations—such as 17q22 amplification—have been linked to enhanced cisplatin sensitivity due to impaired DNA repair. Other mutations (e.g., TP53, PIK3CA/AKT pathway, NF1, PTEN, CHEK2) are associated with progression or resistance and may offer future therapeutic targets, although most are still under clinical investigation.

Although promising, ctDNA is not yet fully validated as a clinical tool in early TNBC. Prospective, multicenter trials (e.g., BRE12-158) are needed to confirm its prognostic and predictive utility across subtypes. Notably, ctDNA outperforms CTCs in early TNBC, though CTCs may retain relevance in other breast cancer types (67). DNA methylation also plays a critical role in the regulation of gene expression in TNBC, contributing to tumor progression and metastasis. In a recent study, methylation alterations were identified in 16 out of 38 TNBC-specific genes and were linked to changes in expression and lymph node involvement. These findings suggest that epigenetic profiling may uncover novel biomarkers for prognosis and guide the development of targeted strategies in this aggressive breast cancer subtype (68).

Overall, the integration of serial ctDNA monitoring into the neoadjuvant management of TNBC holds considerable promise for enhancing risk stratification and enabling timely, personalized interventions. Persistent or re-emerging ctDNA after therapy—regardless of pCR—has been consistently associated with an increased risk of early relapse, underscoring its prognostic value beyond conventional markers. As such, ctDNA represents a powerful tool to refine post-treatment surveillance and guide therapeutic decision-making. Future clinical trials should focus on evaluating ctDNA-informed treatment escalation strategies, particularly for molecular non-responders, in order to improve long-term disease control and survival outcomes in this high-risk population (69–71).

## Conclusions

Liquid biopsy represents a promising, non-invasive tool for monitoring treatment response and disease progression in TNBC. Techniques such as ctDNA and CTCs have demonstrated potential for early detection, prognosis, and therapy guidance, especially in identifying residual disease and predicting recurrence. Notably, ctDNA mutations, including TP53 and BRCA1, may serve as prognostic biomarkers in early-stage TNBC. However, the clinical utility of these biomarkers remains limited by technological variability, lack of standardization, and insufficient large-scale validation. Future efforts must focus on refining detection methods, conducting robust clinical trials, and integrating multi-parametric liquid biopsy approaches—including RNA, exosomes, and immune biomarkers—into personalized treatment strategies to improve patient outcomes in TNBC.

## Limitations and future directions

While liquid biopsy represents a transformative approach for the management of triple-negative breast cancer (TNBC), several limitations currently hinder its widespread clinical integration. First, the sensitivity of ctDNA and CTC detection can be compromised in patients with minimal residual disease or low tumor burden, potentially resulting in false-negative results. Conversely, false positives may arise from clonal hematopoiesis of indeterminate potential (CHIP) or other non-tumor-derived alterations, necessitating careful interpretation of results.

Heterogeneity in assay platforms, pre-analytical handling, and bioinformatic pipelines further complicates cross-study comparisons and limits reproducibility. In TNBC specifically, high intra- and inter-patient heterogeneity poses an additional challenge, as a single liquid biopsy sample may not fully capture the evolving tumor genomic landscape. Furthermore, most of the available data derive from small, retrospective, or early-phase studies, which may be subject to selection bias and lack long-term follow-up.

To address these constraints, future research should prioritize prospective, large-scale, and longitudinal studies with standardized methodologies, integrating multi-omic liquid biopsy analyses and correlating them with robust clinical endpoints. The incorporation of artificial intelligence–driven algorithms to interpret complex, multi-analyte datasets could enhance predictive accuracy and facilitate real-time therapeutic decision-making. Additionally, cost-effectiveness analyses will be critical to ensure equitable access and adoption in routine oncology practice.

Ultimately, overcoming these limitations will be essential to fully realize the potential of liquid biopsy as a reliable diagnostic, prognostic, and treatment-monitoring tool in TNBC.
